# Evaluation of the Real-Life Efficacy and Safety of the Treatment with Lutetium-177 Dotatate for Metastatic Neuroendocrine Tumors

**DOI:** 10.3390/jcm14072384

**Published:** 2025-03-30

**Authors:** Sara Elena Campos Ramírez, Alejandro Andrés García, Carmen Blanco Abad, Paula Gomila Pons, Pablo Gómez Mugarza, Sofía Elena Ruffini Egea, Luis Gallart Caballero, Eduardo Polo Marques, Vicente Alonso Orduña

**Affiliations:** 1 Department of Medical Oncology, Hospital Universitario Miguel Servet, 50009 Zaragoza, Spain; 2 Department of Nuclear Medicine, Hospital Clínico Universitario Lozano Blesa, 50009 Zaragoza, Spain

**Keywords:** lutetium treatment, unresectable neuroendocrine tumors, survival analysis, efficacy of lutetium, safety of lutetium

## Abstract

**Background:** Therapy using lutetium-177 dotatate (^177^LU) was approved in Europe for the treatment of advanced neuroendocrine tumors (NETs) in 2017. Since then, it has become part of the strategies in the treatment of NETs, making it now possible to evaluate real-life results. **Research Design and Methods:** Single-arm, retrospective, multicenter, cohort study of all the patients with metastatic NETs treated with ^177^LU (four cycles of 200 mCi every 8 weeks) in the two medical centers dedicated to the treatment of NETs from the region of Aragón, Spain, from 2017 to 2024. Descriptive analysis of demographic characteristics, efficacy, and survival analysis were performed using the statistics software Jamovi 2.6.14. **Results:** Sixty-eight patients were included. The majority were male, and the most frequent primary location was the pancreas. The ORR was 30.9%. The DCR was 88%. The median OS was 47.4 months [95% CI, 25.6–NE]. The median PFS was 26.1 months [95% CI, 18.5–68.3]. High-grade tumors, multiple previous treatments, and pancreatic location presented worse OS. In total, 42.6% presented any grade adverse event (17.2% hematologic, 30.9% GI symptoms). **Conclusions:** The efficacy of ^177^LU in our study is like that observed in similar studies. Acceptable tolerance has been shown. Pancreatic tumors, previous treatments, and higher grades demonstrated worse outcomes. The new research line must consider the use of treatment with ^177^LU in earlier lines for metastatic disease as well as its possible use in local or locally advanced disease.

## 1. Introduction

Neuroendocrine neoplasms (NENs) are a heterogeneous group of tumors that derive from neuroendocrine cells that are widely distributed in the human body [1]. NENs include neuroendocrine tumors (NETs) and neuroendocrine carcinomas (NECs). The vast majority of NENs are well to poor differentiated NETs.

In Europe, the incidence of NETs ranges between 1.33 and 2.33 per 100,000 people [2]. The most frequent locations include the gastrointestinal (GI) tract (35–55%) as follows: the small intestine (30.8%), rectum (26.3%), colon (17.6%), pancreas (12.1%), and appendix (5.7%). The most frequent extra-gastrointestinal location is the lung [1,3]. At diagnosis, most patients with NETs present localized disease, and around 27% have distant metastases [3].

NETs are classified according to the WHO 2019 classification, which considers parameters such as histological grade (G1, G2, and G3), mitotic count, and Ki-67 index (expressed in percentage). These considerations result in four main groups: well-differentiated NETs (wdNETs) G1, wdNETs G2, wdNETs G3, and poorly differentiated NETs (pdNETs) [4].

For localized disease, surgery is the main curative treatment; regardless, more than 50% of patients with digestive NETs undergoing resection will recur, mostly in distant organs [5].

The greatest prognostic factor is the existence of metastatic disease; patients with metastatic disease have a 10-year prognosis survival between 0% and 30% depending on the primary site location. Other factors, such as poor tumor cell differentiation and pancreatic origin, also present worse outcomes [6].

For metastatic disease, systemic therapy is crucial in controlling tumor growth and symptom development. The standard therapies include somatostatin analogs, interferon, cytotoxic chemotherapy, and other agents such as mTOR inhibitors and tyrosine kinase inhibitors (TKIs) [6].

Somatostatin receptors (SSRs) are considered favorable molecular targets in NETs. A novel type of treatment consists of radiolabeled somatostatin analogs (rSSAs);these are part of the peptide receptor radionuclide therapy (PRRT). PRRT allows the delivery of targeted radiation to tumoral cells by binding a radionuclide to a peptide molecule and then to a specific protein expressed on the cell surface. In the case of rSSA, the peptide is a somatostatin receptor agonist targeted to the somatostatin receptor in tumoral cells [7].

Patients with SSRs positive NETs and normal kidney and bone marrow function are good candidates for PRRT. ^177^LU has been proven as a safe and effective therapy for unresectable and metastatic NETs. When compared with high-dose octreotide, it has been shown to improve the risk of progression or death by about 79% in patients who have progressed to long-acting octreotide [7,8].

Since 2017, this treatment has become one of the options beyond the first line in Spain. Several retrospective studies have been conducted, primarily focusing on the European population, though only a few have included the Spanish population, and these studies have had short follow-up periods. This study aims to evaluate the long-term real-life outcomes of ^177^LU treatment in our region.

## 2. Materials and Methods

### 2.1. Study Design

We conducted a retrospective, whole population, single-arm, multicenter, cohort study. This decision was taken due to the low prevalence of these types of tumors and, therefore, the low number of patients receiving treatment with ^177^LU (Lutathera by Novartis, Montreal, QC, Canada).

### 2.2. Population

Adult (≥18 years old) patients with metastatic NETs, who received at least one session of treatment with ^177^LU (, in the period from January 2017 to November 2024 in any of the two medical centers specialized in NETs treatment (University Hospital Miguel Servet and Clinical University Hospital Lozano Blesa) from the region of Aragón, Spain, according to regional indication as follows:

Patients with metastatic disease with an expression of SSRs in at least 90% of the tumoral lesions, measured by octreotide scan and uptake level of at least 2 indicated by the Krenning score (ranging from 1 to 4). Initial enrollment was performed with an octreotide scan. In recent months, evaluation with PET/CT 68Ga DOTATOC has been introduced to clinical practice. Only a few of these cases were included in the study [n = 7].

The treatment care protocol was one up to four cycles of ^177^LU 200 mCi intravenously administered every 8 weeks (if no contraindications were present in prior blood testing and physician’s evaluation). After each dose of ^177^LU, a lutetium scintigraphy was performed within 24 h to ensure radiopharmaceutical uptake.

Response evaluation with computed tomography with contrast (if no allergies were reported) was performed every two cycles and evaluated according to RECIST 1.1 criteria. This assessment was carried out according to institutional care protocols as part of standard clinical practice, and no additional blood analyses, imaging, or other tests were performed specifically for this study.

Toxicity grading was performed according to the Common Terminology Criteria for Adverse Events (CTCAE).

### 2.3. Data Collection

An original database, which did not include any study variables, was compiled from the list of requests submitted to the Evaluation of Drugs and Sanitary Products in Specialized Attention Committee of Aragón (CEMAE, acronym in Spanish) from the first approval in the region (January 2017) through November 2024. With this solicitude, every single case is evaluated anonymously by a group of members of CEMAE, who decide whether to approve treatment administration in cases where this treatment is not part of international indication; it is evaluated as off-label use for patients with no other treatment options.

From this original database, patients who did not receive at least one full treatment cycle were systematically excluded, resulting in the creation of a refined second database for further analysis.

Information about patient demographics, tumor characteristics, number of cycles of ^177^LU, adverse events, and relevant dates for further survival analysis were obtained from the investigators through the medical history review of each patient included, accessed online from the corresponding centers that participate in this study.

Any identifiable information was meticulously deleted from the final database to ensure complete confidentiality and prevent any type of unauthorized exteriorization of this sensitive information beyond the corresponding center. This rigorous data protection measure was carefully performed for each patient immediately after the completion of data collection and the initial phase of analysis.

### 2.4. Data Analysis

Data were collected and organized using Microsoft Excel, ensuring accuracy and consistency. For comprehensive statistical analysis, both Microsoft Excel and the specialized statistical software Jamovi 2.6.14 were utilized.

A thorough descriptive analysis was conducted to examine the demographic and clinical characteristics of the study population. Additionally, an initial evaluation of treatment efficacy was performed, as well as an evaluation of the safety of the treatment. Key statistical measures, including medians and frequency distributions, were obtained to summarize the data effectively.

To further assess treatment efficacy and explore potential prognostic clinical factors, an in-depth survival analysis was carried out. Kaplan–Meier survival curves were generated to illustrate overall survival (OS) and progression-free survival (PFS) for the entire study group. Subgroup analyses were also performed to identify variations within specific patient categories. When comparisons were required between two or more groups, the Log-Rank test was applied to determine statistical significance with a confidence interval of 95%, and a univariate and multivariate analysis was performed in order to identify possible prognostic factors calculating the Odds Ratio (OR) and Hazard Ratio (HR), respectively.

### 2.5. Ethical Approval

This study was conducted with the official approval of the Investigation Ethics Committee from the Autonomy Community of Aragón (Comité de Ética de la Investigación de la Comunidad Autónoma de Aragón, CEICA, by its Spanish acronym). The requirement for informed consent was waived, as the data utilized in this study had already been documented in patients’ clinical histories solely for routine medical care purposes. Ethical approval for this research was formally registered under the report code EOM25/004, in 15 January 2025.

## 3. Results

### 3.1. General Description Analysis

A total of 68 patients were included in the formal analysis. The median age of the study population at the time of diagnosis was 57 years, with an age range spanning from 20 to 82 years. The majority of the patients were male, accounting for 64.7% of the cohort. The most frequent location of the primary tumor was the gastrointestinal system, representing 70.6% of cases, including tumors originating in the pancreas. All patients had undergone at least one prior line of treatment before receiving **^1^**^77^LU therapy. Metastatic disease most commonly affected the liver, followed by involvement of the lymphatic nodes, lungs, and peritoneum. The median number of cycles of ^177^LU received was four (with a range from one to four), and the median duration of treatment was 170 days ranging from 20 days to 267 days. Additional and more detailed demographic characteristics of the study population are presented in Table 1.

### 3.2. Efficacy Analysis

The overall response rate (ORR) was 30.9% [n = 21], which included patients who exhibited either a complete or partial response to the ^177^LU treatment. Additionally, the disease control rate (DCR) was 88.1% [n = 59], encompassing patients who achieved a complete response, a partial response, or experienced stabilization of the disease without further progression. These findings highlight the therapeutic efficacy of ^177^LU treatment in managing the disease and improving patient outcomes.

Just one patient achieved a complete response to treatment, and the primary location for this case was the pancreas. In total, 29.4% [n = 20] of the patients achieved a partial response, 55.9% [n = 38] showed stabilization of the disease, and 11.8% [n = 8] had progression of the disease during treatment with ^177^LU. One patient died before any possible evaluation of response to treatment. More details about the distribution of the responses according to primary tumor location are shown in Table 2. The percentages shown were calculated in respect of the overall population [n = 68].

**Figure 1:**
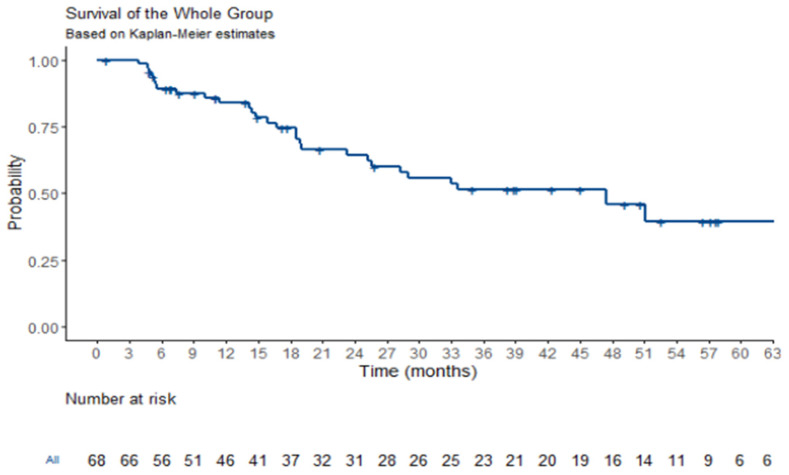
**Overall survival analysis.** Kaplan–Meier survival plot for overall survival for the general population of study. Crosses represent death cases. Median OS was 47.4 months [CI 95%, 25.6–NE]. Twelve-month survival is 84% [75–93.6%, 95% CI]. Thirty-six-month survival is 52% [40–67.3%, 95% CI]. Sixty-month survival is 39% [27–57.4%, 95% CI].

**Figure 2:**
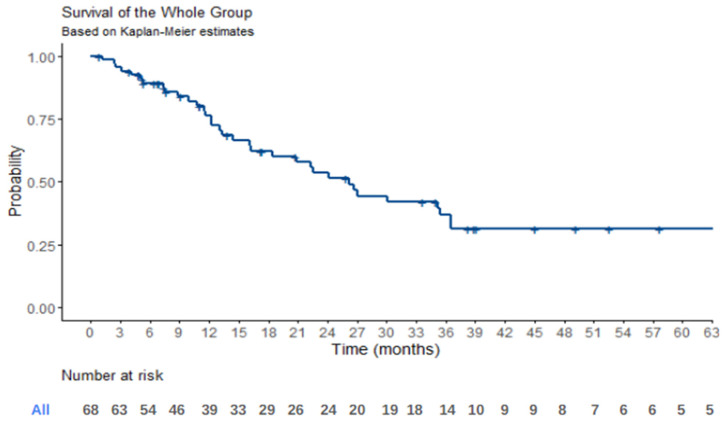
**Progression Free Survival.** Kaplan–Meier survival plot for progression free survival for the general population of study. Crosses represent progression cases. Median PFS was 26.1 months [18.5–68.3, 95% CI]. Twelve-month survival is 76.2% [65.9–88.0%, 95% CI]. Thirty-six-month survival is 36.7% [25.1–53.8%, 95% CI]. Sixty-month survival is 31.5% [20.3–48.7%, 95% CI].

Regarding the survival analysis for the entire patient cohort, at a median follow-up period of 19 months, the median overall survival (OS) was estimated to be 47.4 months [95% confidence interval (CI), 25.6–not estimable]. Additionally, the median progression-free survival (PFS) was determined to be 26.1 months [95% CI, 18.5–36.6]. Kaplan–Meier survival curves illustrating the OS for the whole population are presented in Figure 1, while Figure 2 provides a graphical representation of the PFS data. These survival analyses offer valuable insights into the long-term prognostic outcomes of patients undergoing ^177^LU treatment.

**Figure 3:**
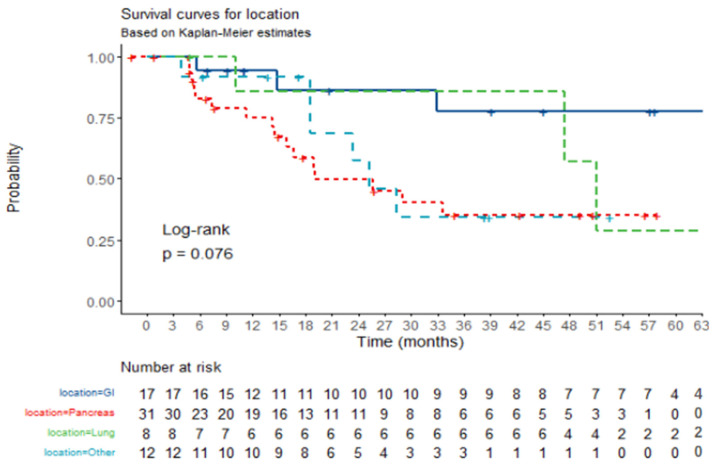
**OS by location of primary tumor.** Kaplan–Meier survival plot for overall survival according to primary tumor location. Crosses represent death cases.When the location is GI, twenty four-month survival is 86.27% [70.08–100%, 95% CI]. When the location is Pancreas, twenty four-month survival is 49.67% [33.37–74%, 95% CI]. When the location is Lung, twenty four-month survival is 85.71% [63.34–100%, 95% CI]. When the location is Other, twenty four-month survival is 57.29% [32.62–100%, 95% CI].

When analyzed by subgroup, pancreatic tumor location appears to be associated with worse OS compared to tumors originating in other locations (GI tract, lung, and other locations). Specifically, patients with pancreatic tumors had a Hazard Ratio (HR) of 3.7 [95% CI, 1.20–11.43; *p* = 0.022] when compared to those with tumors in the GI tract, which emerged as the primary location associated with better survival outcomes. The median OS for pancreatic location was calculated to be 19 months [95% CI, 15.9–NE], and for GI location, it was 65.6 months [95% CI, 65.7–NE]. This suggests that tumor origin might play a significant role in patient prognosis. The Kaplan–Meier survival plot illustrating these differences is presented in Figure 3.

**Figure 4:**
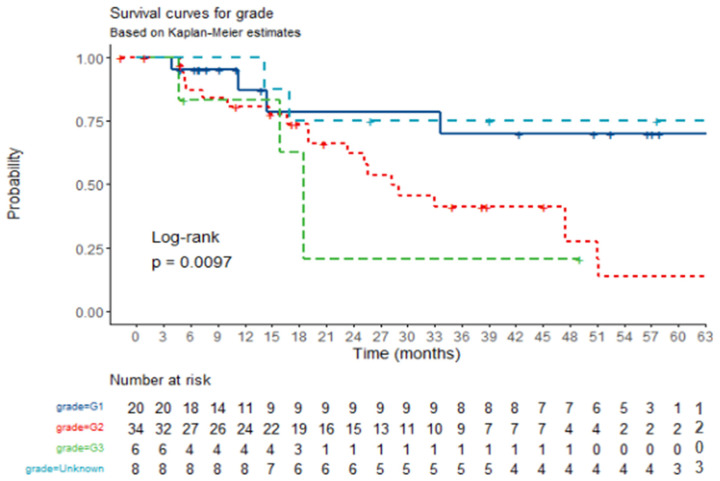
**OS by histological grade.** Kaplan–Meier survival plot for overall survival according to histological grade. Crosses represent death cases.When the histological grade is G1, twenty four-month survival is 78.4% [58.9–100%, 95% CI]. When the histological grade is G2, twenty four-month survival is 61.9% [46.3–83%, 95% CI]. When the histological grade is G3, twenty four-month survival is 20.8% [3.7–100%, 95% CI]. When the histological grade is Unknown, twenty four-month survival is 75.0% [50.3–100%, 95% CI].

Patients with higher-grade tumors (G3 and G2) demonstrated significantly worse OS compared to those with low-grade (G1) tumors. The Kaplan–Meier survival plot illustrating these differences in survival among tumor grades is presented in Figure 4.

**Figure 5:**
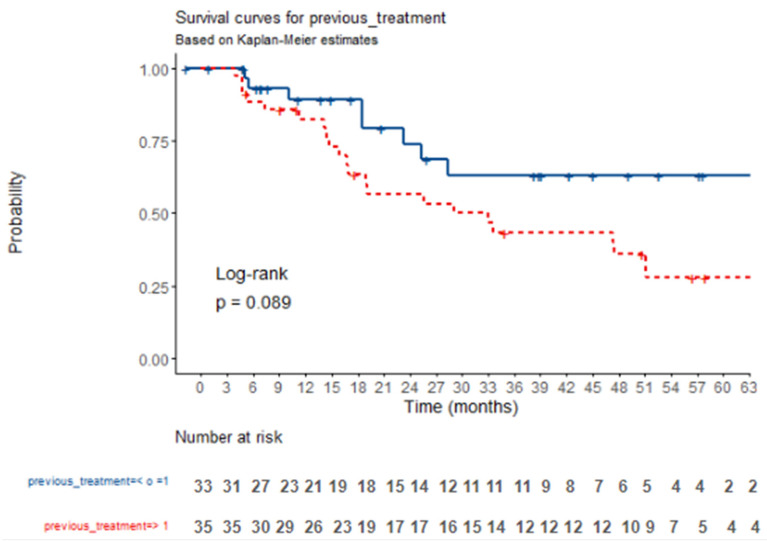
Kaplan–Meier survival plot for overall survival according to the number of previous treatments received. Crosses represent death cases. When previous treatment is < o =1, twenty four-month survival is 74% [58–95%, 95% CI]. When previous treatment is > 1, twenty four-month survival is 57% [42–77%, 95% CI].

All patients included in our study had been treated with at least one systemic therapy before receiving ^177^LU treatment and had experienced disease progression. A total of 48.5% [n = 33] of patients received ^177^LU therapy as a second-line treatment, while the remaining 51.4% [n = 35] had undergone two or more lines of treatment before initiating ^177^LU therapy. The median number of prior treatments was two, with a range spanning from one to seven. Notably, patients who have received more than one prior systemic treatment exhibited worse OS compared to those who have only undergone a single previous therapy, with an HR of 1.94 [95% CI, 0.89–4.22; *p* = 0.095]. The median OS for patients with just one prior therapy was 65.6 months [95% CI, 28.3–NE] and 32.9 months [95% CI, 16.9–NE] for patients with two or more prior treatments. Further details and survival analysis are illustrated in Figure 5.

A univariate survival analysis was performed concerning these same three variables (histological grade, location of primary tumor, and number of previous treatments received), with the Odds Ratio (OR) being statistically significant for histological grade (favoring G1 and unknown) and the number of previous treatments received (favoring ≤1 previous treatments), but not for location (Table 3).

The multivariate survival analysis concerning histological grade and the number of previous treatments appears to confirm that these two are poor prognostic factors, for histological grade also yielding statistically significant differences. The details of this second analysis are provided in Table 4.

### 3.3. Safety Analysis

Adverse events (AEs) of any type were reported in 42.6% [n = 29] of patients in our study population. Toxicity grading was performed according to the Common Terminology Criteria for Adverse Events (CTCAE). The most frequently observed AE was digestive toxicity (nausea, vomiting, and/or diarrhea), affecting 30.9% [n = 21] of patients, followed by hematological toxicity (leukopenia and/or thrombopenia), which was documented in 17.2% [n = 11], and generalized asthenia in 5.9% [n = 4]. The only grade 3/4 AEs reported were white blood cell decreased [n = 1] and platelet count decreased [n = 2]. Importantly, no treatment-related mortality was observed, indicating that ^177^LU therapy was well tolerated. However, a subset of patients [8.8%, n = 6] were unable to complete the full treatment regimen due to inadequate recovery from AEs. Among these patients, only two were required to discontinue treatment prematurely because of renal toxicity (creatinine increased levels, both grade 2). In contrast, the remaining four had to terminate the therapy due to hematologic toxicity.

A comprehensive breakdown of treatment-related toxicities is provided in Table 5.

## 4. Discussion

### 4.1. Population Characteristics

In our study, the majority of patients were male, and the most frequently observed location of the primary tumor was the pancreas. Given that our study specifically included only patients with metastatic disease, these findings align with previously reported observations in the literature [9]. In cases of localized and locoregionally advanced disease, the most commonly observed characteristics include a higher prevalence among women and a primary tumor location within the digestive tract [1,10]. Other demographic characteristics such as median age and site of metastasis are along with observed in multiple studies.

### 4.2. Efficacy of Treatment

Regarding the evaluation of the efficacy of treatment with ^177^LU, extensive research has been conducted through multiple clinical trials. Early phase I/II studies involve cohorts ranging from 35 to 51 patients with unresectable gastroenteropancreatic (GEP) and bronchial NETs. These patients underwent treatment with ^177^LU, and the results obtained led to the establishment of a standard protocol consisting of four cycles, each administered with a dosage of 200 mCi of ^177^LU intravenously every 8 weeks, for up to fourcycles. Preliminary efficacy data were promising, with a disease control rate (DCR) ranging from 69% to 88%, with acceptable tolerance [11].

These studies led to the development of the phase III clinical trial NETTER-1, which included 231 patients with unresectable or metastatic well-differentiated midgut NETs, who were randomly assigned to be treated with four cycles of 200 mCi every 8 weeks of ^177^LU versus 60 mg of octreotide every 4 weeks. After a median follow-up of 76.3 months, the median OS was 48.0 months (95% CI: 37.4–55.2) in the ^177^LU arm. The ORR was 37%, and the DCR was 78% [12].

Although the NETTER-1 represents a major scientific reference that led to the approval of this treatment in patients with NET after at least one line of systemic treatment, we consider that our findings cannot be contrasted with this study, since no patients with pancreatic NETs were included in the NETTER-1 trial, and they represent the majority of patients in our study population.

The most robust efficacy data for ^177^LU treatment in pancreatic NETs has been reported in the NETTER-R study, a phase IV, observational, post-authorization retrospective registry study. Among the 62 patients with measurable pancreatic tumors assessed, the median PFS was 24.8 months [95% CI, 17.5–34.5], while the median OS was 41.4 months [95% CI, 28.6–50.2]. The median OS and PFS observed in our study (47.4 and 26.1 months, respectively) are highly comparable. However, the ORR reached 40.3%, whereas the ORR in our study (30.9%) is lower than that reported in the NETTER-R study [13].

In a study conducted by Brabander T et al. [14], a total of 1214 Dutch patients with NETs originating from the gastrointestinal (GI) tract, pancreas, lungs, or an unknown primary site were treated with ^177^LU over a period of five years, starting in January 2000. A subgroup analysis of the 443 patients who received at least 600 mCi of ^177^LU reported an ORR of 39% and a DCR between 81% and 84%, a median OS of 46 months [95% CI, 32–60 months], and a median PFS of 24 months [95% CI, 18–30 months] [14]. The median OS and PFS in our study (47.4 and 26.1 months, respectively) are highly comparable to those reported in this study. The DCR (88%) is also in line with the findings from this study. Once again, the ORR is slightly higher than that observed in our study.

### 4.3. Prognostic Factors

Further survival analysis across different subgroups in our study and univariate and multivariate analysis suggests that location might be a significant prognostic factor, with poorer outcomes when the primary site is the pancreas and better outcomes for GI locations. This is in accordance with their more aggressive course with metastases to the liver as well as other distant sites. The presence, progression, and expansion of liver metastases are linked to poor prognosis and have been reported in up to 80% of pancreatic NET patients in certain studies [15].

A similar phenomenon is observed in the histological grade subgroup analysis, where higher grades seem to have worse outcomes. This finding suggests that tumor differentiation plays a crucial role in patient prognosis, with poorly differentiated and moderately differentiated tumors being associated with more aggressive disease progression and reduced survival outcomes. In the NETTER-2 study, a randomized phase III study that included 226 patients with newly diagnosed GEP NETs grade 2 or 3 from North America, Europe, and Asia, after formal analysis, a lower median PFS of 22.8 months [CI95%, 19.4–NE] was reported [16]. This is, as well, the first study to test ^177^LU treatment as a first-line option.

These findings are consistent with the results from another cohort study by Zhang J et al. [17], which included 69 patients diagnosed with grade 3 wdNETs who were treated with either ^177^LU or ^90^Y-labeded somatostatin analogs. In this study, 46 patients had pancreatic location as the primary tumor site. The median OS was 19.9 months, and the median PFS was 9.6 months [17].

In our study, we observed that patients who have been treated with ^177^LU as a second-line option compared with patients who received this treatment in further lines seemed to have better results. This trend suggests that a greater number of prior treatments may be associated with more advanced and poorer prognosis. Apart from the NETTER-2 study, there is currently no substantial evidence supporting the use of ^177^LU therapy as a first-line treatment option.

### 4.4. Safety of Treatment

Respecting the safety of the treatment with ^177^LU, in the initial analysis of data from the NETTER-1 trial in 2017, the most frequently reported AEs were nausea (59%) and vomiting (47%). These side effects were primarily attributed to the amino acid infusions administered simultaneously with ^177^LU therapy, as these events were transient and resolved once the infusions were completed [12]. The incidence of these AEs has been reduced using premedication with antiemetic drugs administered prior to the infusion of the amino acid solution, improving tolerance to treatment.

In our study as well as in other studies reported so far, the incidence of digestive symptoms is lower, probably due to the premedication administered before each cycle of ^177^LU.

In a recent analysis by Strosberg J et al. [18], based on symptom diaries of patients enrolled in the NETTER-1 trial, ^177^Lu therapy was not only well tolerated both during and after infusions but was also associated with a significant improvement in quality of life. This benefit was achieved by effectively reducing symptoms related to NETs, highlighting its potential therapeutic value beyond tumor control [18].

The second most frequently observed AEs were hematologic toxicities, which are consistent with previously reported data, showing an incidence of less than 20% for each type of blood cell decrease [11]. It is worth noting that although hematologic toxicity is not alarmingly frequent, it remains the leading cause of treatment discontinuation in patients receiving ^177^Lu therapy, underscoring the need for careful monitoring and management.

### 4.5. Limitations of the Study

The main limitations of our study stem from its design; it is a retrospective study, and it lacks a control arm for comparison with ^177^LU therapy, making it a non-randomized study. However, our patient population shares similar characteristics with other real-world retrospective studies, reflecting everyday clinical practice. Many patients had received prior treatment, some of them being heavily treated, and had tumors with poor prognostic factors, such as pancreatic primary location, liver metastases, and high-grade tumors. Despite these challenges, treatment with ^177^LU appears to yield promising and encouraging results, improving the quality of life and survival of patients affected by metastatic NETs.

Enrollment in treatment and evaluation of disease was performed within the standard care protocol from our region; this has been changing throughout the years.

Initial enrollment was initially performed with an octreotide scan. In the recent months, evaluation with PET/CT 68Ga DOTATOC was introduced to clinical practice. Only a few of these cases were included in the study. This fact, along with recent studies that suggest there could be a mismatch between different imaging techniques, could have led to losses of patients in real life [19].

### 4.6. Further Lines of Investigation

In a meta-analysis by Strosberg J et al. [20], re-treatment with ^177^LU showed a median PFS of 12.52 months [95%CI, 9.82–15.22]. This study included 414 patients from 13 studies who were previously treated with ^177^LU and/or ^99^Y-PRRT (Yttrium-90 (^90^Y)-based PRRT) and progressed to it. In patients with progressive GEP NETs who were treated with ^177^LU, PFS from the first treatment with PRRT ranged from over 12 to 18 months. The safety profile was very similar to that reported in initial PRRT treatment studies, with a slightly higher hematologic toxicity. In our population, there were a few patients who were re-treated with ^177^LU, but since the total number was low, they were not analyzed in this study [20].

On the other hand, as mentioned above, there are initial data from the NETTER-2 trial, that support the usage of treatment with ^177^LU as a first-line therapy in patients with grade 2 and 3 NETs.

The addition of capecitabine or temozolomide during treatment with ^177^LU has also been studied by Swayamjeet et al. [21] and Sander M. et al. [22], respectively. Promising results in ORR and DCR indicate these combinations could be an interesting line of study, as they could enhance tumor uptake of radioactivity [21,22].

Another potential line of study is to add treatment with ^177^LU in earlier-stage disease. In 2021, Parghane et al. [23] conducted a prospective study with patients with unresectable GEP NETs, with or without potentially resectable liver metastases. After four to five cycles of ^177^LU standard dose, 15 out of 57 patients became resectable and underwent complete surgery of the primary tumor and liver metastases [23].

The NEOLUPANET was a single-arm, phase II trial of 31 patients with resectable or potentially resectable non-functioning pancreatic NETs, treated with neoadjuvant ^177^LU up to four cycles before complete resection. Partial response was observed in 18 out of 31 patients, and no disease progression was observed. Authors conclude that neoadjuvant ^177^LU is safe and effective for patients with non-functioning pancreatic NETs. Further data on long-term outcomes are not available [24].

## 5. Conclusions

The findings in our study are similar to those observed in other studies. However, considering that our population included only metastatic NETs, and the major representation of liver metastases originating from the pancreas, ORR is slightly lower than observed in other studies. In any case, median PFS, OS, and DCR are very similar to those reported so far.

There is strong evidence that therapy with ^177^LU is safe for the treatment of GEP metastatic tumors and does not affect the quality of life of patients.

Pancreatic location, multiple previous treatments, but specifically higher grades seem to be worse prognosis factors. Considering that from these factors, the only modifiable one is to provide treatment with ^177^LU in earlier lines, and with the actual evidence, we think treatment with ^177^LU as first-line for metastatic disease must be considered.

The usage of ^177^LU in earlier-stage disease must be studied more in-depth, and it could be considered for very selected patients.

## Figures and Tables

**Table 1:** Demographic characteristics.

Characteristic	Overall (n = 68)	Cumulative %
Gender		
Male	44 (64.7%)	64.7%
Female	24 (35.3%)	100%
Primary tumor location		
GI tract	17 (25.0%)	25.0%
Pancreas	31 (45.6%)	70.6%
Lung	8 (11.8%)	82.4%
Others	12 (17.6%)	100%
Metastatic location		
Liver	62 (91%)	-
Lungs	5 (7.4%)	-
Peritoneum	14 (20.6%)	-
Bone	26 (38.2%)	-
Previous treatments		
≤1	33 (48.5%)	48.5%
>1	35 (51.5%)	100%
Tumor differentiation		
Well differentiated	59 (86.8%)	86.8%
Moderately differentiated	2 (2.9%)	89.7%
Poorly differentiated	4 (5.9%)	95.6%
Unknown	3 (4.4%)	100%
Grade		
G1	20 (29.4%)	29.4%
G2	34 (50.0%)	79.4%
G3	6 (8.8%)	88.2%
Unknown	8 (11.8%)	100%

N = number, GI = Gastrointestinal.

**Table 2:** Distribution of treatment response by location of primary tumor.

	Location	Frequency	% from Total	Cumulative %
Complete response	GI	0	0.0%	0.0%
	Pancreas	1	1.5%	1.5%
	Lung	0	0.0%	1.5%
	Other	0	0.0%	1.5%
Stabilization of the disease	GI	12	17.6%	19.1%
	Pancreas	14	20.6%	39.7%
	Lung	5	7.4%	47.1%
	Other	7	10.3%	57.4%
Partial response	GI	2	2.9%	60.3%
	Pancreas	11	16.2%	76.5%
	Lung	2	2.9%	79.4%
	Other	5	7.4%	86.8%
Progressive disease	GI	1	1.5%	88.2%
	Pancreas	6	8.8%	97.1%
	Lung	1	1.5%	98.5%
	Other	0	0.0%	98.5%
Unknown	GI	0	0.0%	98.5%
	Pancreas	1	1.5%	100.0%
	Lung	0	0.0%	100.0%
	Other	0	0.0%	100.0%

GI = Gastrointestinal.

**Table 3:** Univariable analysis.

Independent Variable	Category	Yes (n)	No (n)	OR (95%CI)	*p* Value
Histological grade	G1	4	16	Reference value	Reference value
	G2	21	13	6.46 (1.90–26.58)	*p* = 0.005
	G3	4	2	8.0 (1.16–75.78)	*p* = 0.044
	Unknown	2	6	1.33 (0.16–8.94)	*p* = 0.771
Previous treatments	≤1	9	24	Reference value	Reference value
	>1	22	13	4.45 (1.66–13.14)	*p* = 0.004
Location	GI	5	12	Reference value	Reference value
	Pancreas	16	15	3.0 (0.84–12.58)	*p* = 0.105
	Lung	5	3	5.0 (0.85–35.46)	*p* = 0.084
	Other	6	6	3.0 (0.62–16.11)	*p* = 0.178

GI = Gastrointestinal, OR = Odds ratio.

**Table 4:** Multivariable analysis.

Independent Variable	Category	All	HR (95% CI)	*p* Value
Grade	G1	20	Reference value	Reference value
	G2	34	3.41 (1.15–10.07)	*p* = 0.027
	G3	6	7.83 (1.80–34.13)	*p* = 0.006
	Unknown	8	0.82 (0.16–4.62)	*p* = 0.823
Previous treatments	≤1	33	Reference value	Reference value
	>1	35	2.22 (0.97–5.07)	*p* = 0.058

HR = Hazard ratio.

**Table 5:** Safety analysis.

Adverse Event	Frequency/Overall (%)	Cumulative %
Any adverse event		
Yes	29/68 (42.6%)	42.6%
No	3/68 (57.4%)	100%
Digestive		
Nausea	12/68 (17.6%)	-
Grade 1	11/12 (91.7%)	91.7%
Grade 2	1/12 (8.3%)	100.0%
Vomiting	9/68 (13.2%)	-
Grade 1	8/9 (88.9%)	88.9%
Grade 2	1/9 (11.1%)	100.0%
Diarrhea	9/68 (13.2%)	-
Grade 1	9/9 (100%)	100%
Hematologic		
WCC decrease	8/68 (11.8%)	-
Grade 1 Grade 4	7/8 (87.5%) 1/8 (12.5%)	87.5% 100.0%
PC decrease	4/68 (5.9%)	-
Grade 2 Grade 3 Grade 4	2/4 (50.0%) 1/4 (25.0%) 1/4 (25.0%)	50.0% 75.0% 100.0%
General		
Asthenia	4 (5.9%)	-
Grade 1	4/4 (100%)	100%

WCC = White Cell Count, PC = Platelets Count.

## Data Availability

Available upon request to corresponding authors.

## Abbreviations

The following abbreviations are used in this manuscript:

^177^LU	Lutetium-177 dotatate
AEs	Adverse events
CEICA	Ethics Committee from the Community of Aragón
CEMAE	Evaluation of Drugs and Sanitary Products in Specialized Attention Committee of Aragón
CI	Confidence interval
GI	Gastrointestinal
GEP	Gastroenteropancreatic
NECs	Neuroendocrine carcinomas
NENs	Neuroendocrine tumors neoplasms
NETs	Neuroendocrine tumors
ORR	Overall response rate
OS	Overall survival
pd	Poorly differentiated
PFS	Progression free survival
PRRT	Peptide receptor radionuclide therapy
rSSA	Radiolabeled somatostatin analogs
SSAs	Somatostatin analogs
SSRs	somatostatin receptors
wd	Well differentiated
^99^Y-PRRT	Yttrium-90 (^90^Y)-based PRRT
